# Green HPLC-Fluorescence detection method for concurrent analysis of Tamsulosin hydrochloride and Tolterodine tartrate in dosage forms and biological fluids

**DOI:** 10.1038/s41598-025-92183-6

**Published:** 2025-03-27

**Authors:** Sayed M. Derayea, Khalid M. Badr El-Din, Ahmed S. Ahmed, Mohamed Oraby, Mohamed A. Abdelshakour

**Affiliations:** 1 Department of Analytical Chemistry, Faculty of Pharmacy, Minia University, Minia, 61519 Egypt; 2 Department of Pharmaceutical Analytical Chemistry, Faculty of Pharmacy, Sohag University, Sohag, 82524 Egypt; 3 Department of Pharmaceutical Chemistry, College of Pharmacy, Al-Esraa University, Baghdad, 10069 Iraq

**Keywords:** Tamsulosin, Tolterodine, Gradient HPLC elution, Spiked human plasma, Spiked human urine, Green chemistry, Chemistry, Analytical chemistry

## Abstract

A novel, environmentally friendly HPLC-FL method has been developed to simultaneously quantify tamsulosin hydrochloride (TAM) and tolterodine tartrate (TTD) in pharmaceutical formulations. The method effectively separates the drugs using an ODS column and gradient elution at a flow rate of 1.0 mL/min. Fluorescence detection was used to measure the relative fluorescence intensity (RFI) of the analytes, with excitation and emission wavelengths set at 280 nm and 350 nm, respectively. The retention times for TAM and TTD were determined to be 5.66 and 7.26 min, respectively. The method’s accuracy and precision were validated according to ICH guidelines. Linear calibration curves were obtained over the concentration ranges of 0.1–1.5 µg mL^-1^ for TAM and 1–15 µg mL^-1^ for TTD, with correlation coefficients exceeding 0.9995. The calculated LOD values for TAM and TTD were 0.03 and 0.30 µg mL^-1^, respectively. The corresponding LOQ values were 0.10 and 0.92 µg mL^-1^, respectively. The method’s applicability was demonstrated by analyzing pharmaceutical dosage forms and spiked plasma and urine samples. The observed mean recoveries percent were 99.31-100.97% for TAM and 100.56-101.79% for TTD in human plasma, while in human urine, they were 98.71-101.55% for TAM and 99.53-101.84% for TTD. To evaluate the method’s greenness, GAPI and AGREE tools were employed. The results indicated significant adherence to green chemistry principles.

**Supplementary Information:**

The online version contains supplementary material available at 10.1038/s41598-025-92183-6.

## Introduction

The improvement of analytical methods has a significant impact on pharmaceutical analysis. Drug substances and impurities in pharmaceutical ingredients, formulations, and biological matrices must be identified and quantified for numerous applications^1,2^. Because of its ease of use, high sensitivity, and specificity, HPLC has recently drawn a lot of interest from the pharmaceutical and biological fluid industries. For assaying biological matrices, HPLC in conjunction with various detection techniques like ultraviolet (UV), fluorescence, and mass spectrometry is currently the recommended approach^3–5^.

**Fig. 1:**
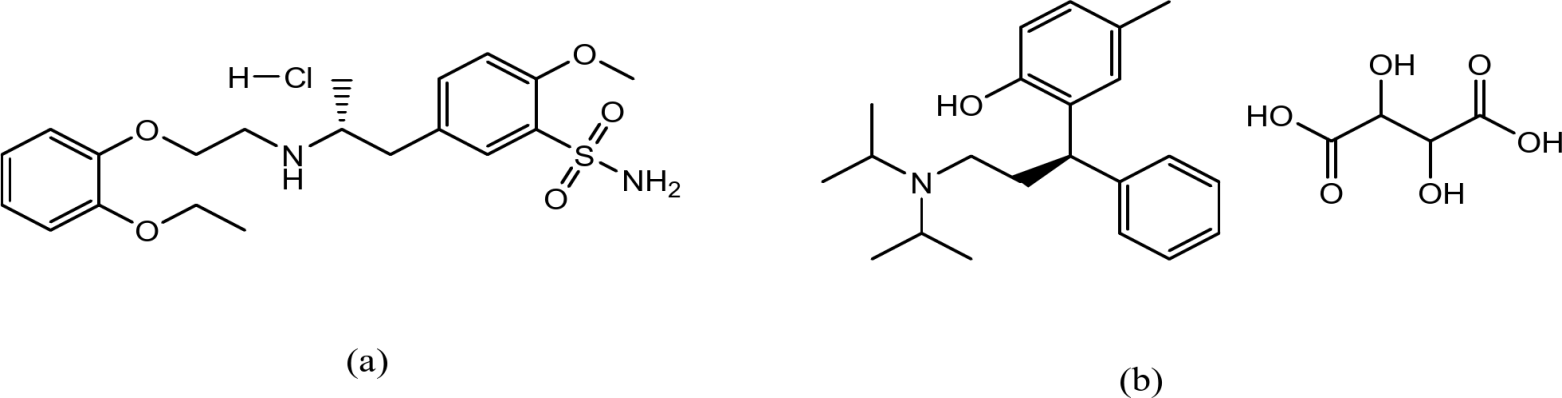
Chemical structures of (**a**) TAM and (**b**) TTD.

Overactive bladder (OAB) is a common disorder among men over 45, harmfully impacting their quality of life. To manage OAB associated with benign prostatic hyperplasia (BPH), healthcare providers often prescribe alpha-blockers^6^. However, recent studies suggest that combining antimuscarinics with alpha-1 blockers may provide more effective relief for BPH-related OAB than using alpha-blockers alone^7^. Tamsulosin hydrochloride (TAM) is a selective alpha-1-blocker used to treat BPH. The chemical structure is illustrated in Fig. 1a. Tolterodine Tartrate (TTD), depicted in Fig. 1b, is an antimuscarinic medication for urinary incontinence. The development of innovative analytical methods is crucial to analyze novel drug combinations like TAM and TTD, used to treat BPH and OAB. TAM is an approved drug in both the United States Pharmacopeia (USP) and the British Pharmacopoeia (BP)^8,9^. The USP describes an HPLC method for analyzing TAM content in capsules^8^. While the BP outlines a potentiometric titration method for quantifying TAM in bulk powder^9^. A comprehensive literature review reveals various techniques for assessing TAM, including spectrophotometry^10^, spectrofluorimetry^11^, HPLC^12^, and voltammetry^13^. TTD is officially recognized as a pharmaceutical substance solely within the BP. The BP details a potentiometric titration method for assessing TTD in bulk form^9^. Various techniques have been reported for analyzing TTD, including spectrophotometry^14^, spectrofluorimetry^15^, HPLC^16^, HPTLC^17^, and voltammetry^18,19^. However, methods for the simultaneous analysis of TAM and TTD are relatively limited, with spectrophotometry^20^, spectrofluorimetry^21^, HPLC-UV^22,23^, and HPTLC^24–26^ approaches for their analysis in pharmaceutical formulations.

Direct spectrophotometric methods have limited sensitivity, while direct spectrofluorimetric methods offer high sensitivity. However, both lack the selectivity of chromatographic methods^27–30^. HPLC with fluorescence detection provides both high sensitivity and selectivity. Consequently, there was a need to create a simple, dependable, rapid, green significantly more sensitive, and selective HPLC with fluorescence detection method for the concurrent analysis of TAM with TTD. Also, the technique would be used to assay them in spiked human plasma and urine. So, it could be used in monitoring the drug’s quality to ensure patient safety^31,32^. The suggested approach was determined to meet the standards of the International Council for Harmonization (ICH)^33^.

## Experimental

### Instrumentations

The HPLC system employed in the experiment consisted of a Sykam S 1130 quaternary pump (Sykam GmbH, Gewerbering, Germany) equipped with an integrated vacuum degasser. A Wakopack Handy-ODS (Wako Pure Chemicals, Osaka, Japan) column (150 × 4.6 mm, 5 μm particle size) was used for chromatographic separation. Fluorescence detection was performed using an RF-20 A fluorescence detector (Sykam GmbH, Gewerbering, Germany). Double-distilled water was used in the experiment, and the pH of the phosphate buffer was adjusted using a Jenway 3510 pH meter (Staffordshire, UK). A Mettler Toledo 5-digit balance (Greifensee, Switzerland) was used for weighing.

### Materials and reagents

The reference standards for TAM (purity of 99.55%) and TTD (purity of 99.70%) were generously provided by Amoun Pharmaceutical Company (El-Obour City, Egypt) and Adwia Pharmaceuticals Company (10th of Ramadan, Sharqia, Egypt), respectively. Tamsul^®^ tablets (0.4 mg TAM) and Incont L.A.^®^ tablets (4 mg TTD), produced by Amoun Pharmaceutical Company and Adwia Pharmaceuticals Company, respectively, were purchased from a local pharmacy. The chemicals used in the experiment, including HPLC-grade methanol, HPLC-grade acetonitrile, disodium hydrogen phosphate, and phosphoric acid were supplied by Merck (Darmstadt, Germany).

Human plasma was generously donated by Sohag University Hospital Blood Bank (Sohag, Egypt). It was kept frozen at −20 °C until the analysis was done.

### Mobile phase and chromatographic conditions

The mobile phase for the HPLC analysis consisted of three solvents: acetonitrile (A), water (B), and phosphate buffer (10 mM, pH 3.0) (C). A gradient elution system was employed, with the specific gradient profile listed in Table 1. All chromatographic experiments were conducted at room temperature with a mobile phase flow rate of 1.0 mL/min. Fluorescence detection was used to measure the relative fluorescence intensity (RFI) of the analytes, with excitation and emission wavelengths set at 280 nm and 350 nm, respectively. The phosphate buffer was prepared by dissolving 1.42 g of disodium hydrogen phosphate in 1.0 L of double-distilled water and adjusting the pH to 3.0 with phosphoric acid. Before injecting samples into the HPLC system, the column was allowed to stabilize for at least 30 min with the mobile phase. An injection volume of 20 µL was used for all experiments.

**Table 1:** Time table of the gradient elution used for chromatographic separation of TAM and TTD.

Time (min)	solvent A (%) (Acetonitrile)	solvent B (%) (Water)	solvent C (%) (Phosphate buffer pH 3)	Flow rate mL/min
Initial	40	60	0	1.0
1	40	60	0	1.0
5.5	50	0	50	1.0
9	80	0	20	1.0
10	40	60	0	1.0

### Preparation of standard solutions

**Fig. 2:**
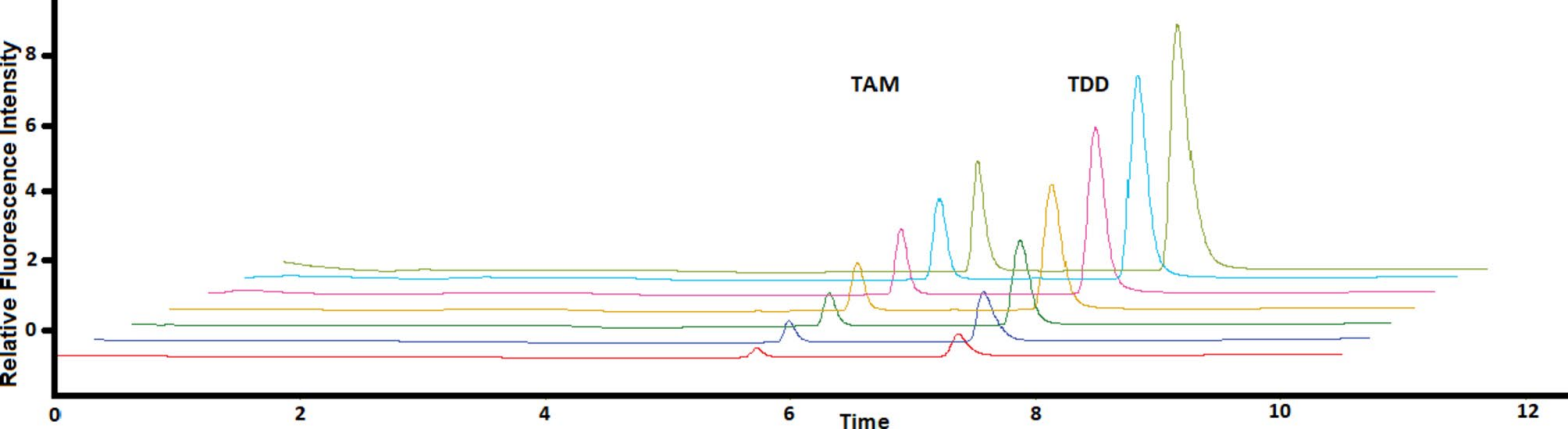
Overlay chromatogram of seven linearity levels for separating TAM and TTD (0.15, 0.3, 0.5, 0.8, 1.0, 1.2, and 1.5 µg mL^−1^ for TAM and 1.5, 3.0, 5.0, 8.0, 10.0, 12.0, and 15.0 µg mL^−1^ for TTD, respectively) using 280 nm and 350 nm as excitation and emission wavelengths, respectively.

Stock standard solutions of TAM and TTD, each at a concentration of 200 µg mL^[-1^, were prepared by dissolving 20 mg of each drug in 100 mL of methanol. These stock solutions were stored at 4 °C. The calibration curve was prepared by further diluting the stock solutions with methanol to obtain the desired concentration ranges, as shown in Fig. 2.

### Procedure for TAM and TTD synthetic pharmaceutical formulation

**Fig. 3:**
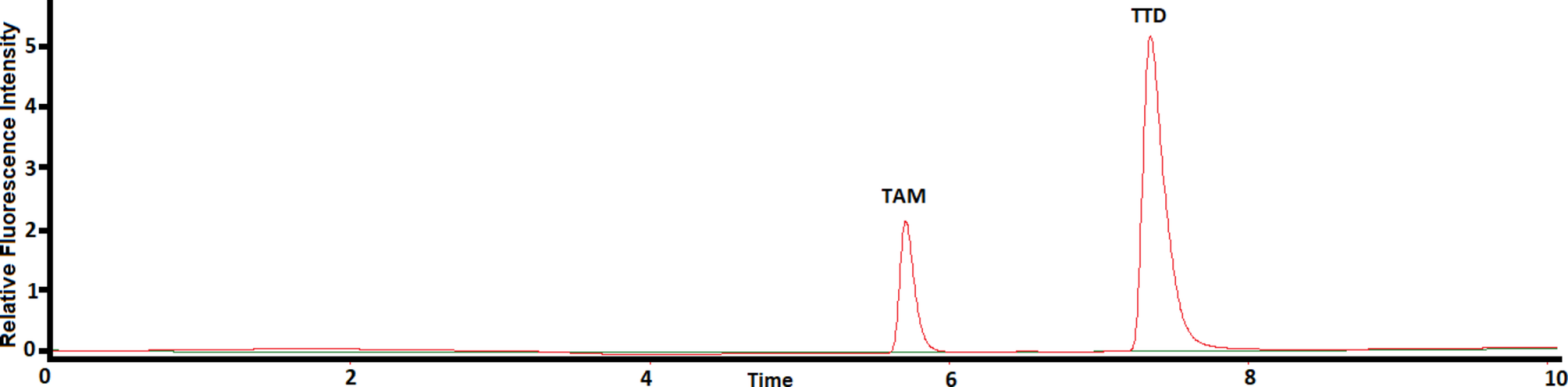
The chromatogram of: (—) the blank and (—) synthetic pharmaceutical formulation containing TAM (1.0 µg mL^−1^) and TTD (10.0 µg mL^−1^) using 280 nm and 350 nm as excitation and emission wavelengths, respectively.

Due to the unavailability of the combined pharmaceutical tablet containing TAM and TTD in local Egyptian pharmacies, a synthetic pharmaceutical formulation was prepared in the laboratory. The process involved weighing and grinding ten Tamsul® tablets, each containing 0.4 mg of TAM, to determine their average weight. Simultaneously, the contents of ten Incont L.A.® tablets, each containing 4 mg of TTD, were weighed and ground to determine the average weight of TTD. A specific amount of powdered tablet, equivalent to 1 mg of TAM and 10 mg of TTD, was dissolved in 70 mL of methanol. The resulting mixture was then transferred to a 100 mL volumetric flask and thoroughly mixed. After 30 min of sonication, the volume of the solution in the flask was adjusted to the mark using methanol. The solution was then filtered, and the initial portion of the filtrate was discarded. Subsequently, 1 mL of the filtered solution was transferred to a 10 mL volumetric flask and diluted to volume with methanol. A representative chromatogram of the sample is shown in Fig. 3.

### Procedure for TAM and TTD in biological human samples

#### Procedure for TAM and TTD in spiked human plasma

**Fig. 4:**
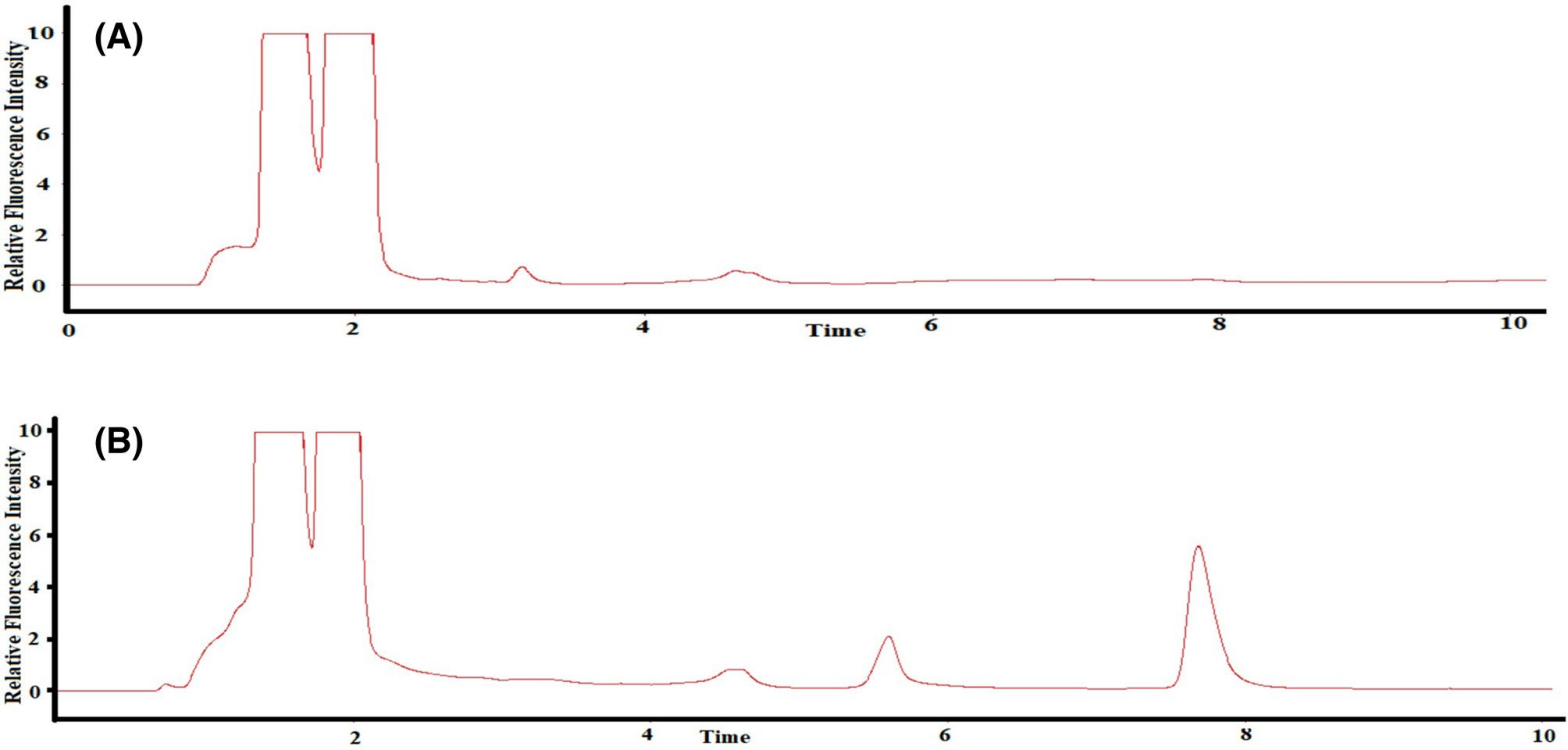
The chromatogram of (**A**) plasma blank and (**B**) plasma containing TAM (1.25 µg mL^−1^) and TTD (12.5 µg mL^−1^) using 280 nm and 350 nm as excitation and emission wavelengths, respectively.

2.0 mL of stored plasma was mixed with 1.0 mL of a standard solution containing TAM and TTD (concentration ranges: 1–15 µg mL^[-1^for TAM and 10–150 µg mL^[-1^ for TTD) and 6.0 mL of methanol was finally added to precipitate plasma proteins. The mixture was vortex-mixed for 60 s and then centrifuged at 4000 rpm for 10 min to separate the precipitated proteins. The clear supernatant, which fell within the linear range of the proposed chromatographic method, was injected into the HPLC system. A blank sample was prepared similarly using drug-free plasma (Fig. 4).

#### Procedure for TAM and TTD in spiked human urine

**Fig. 5:**
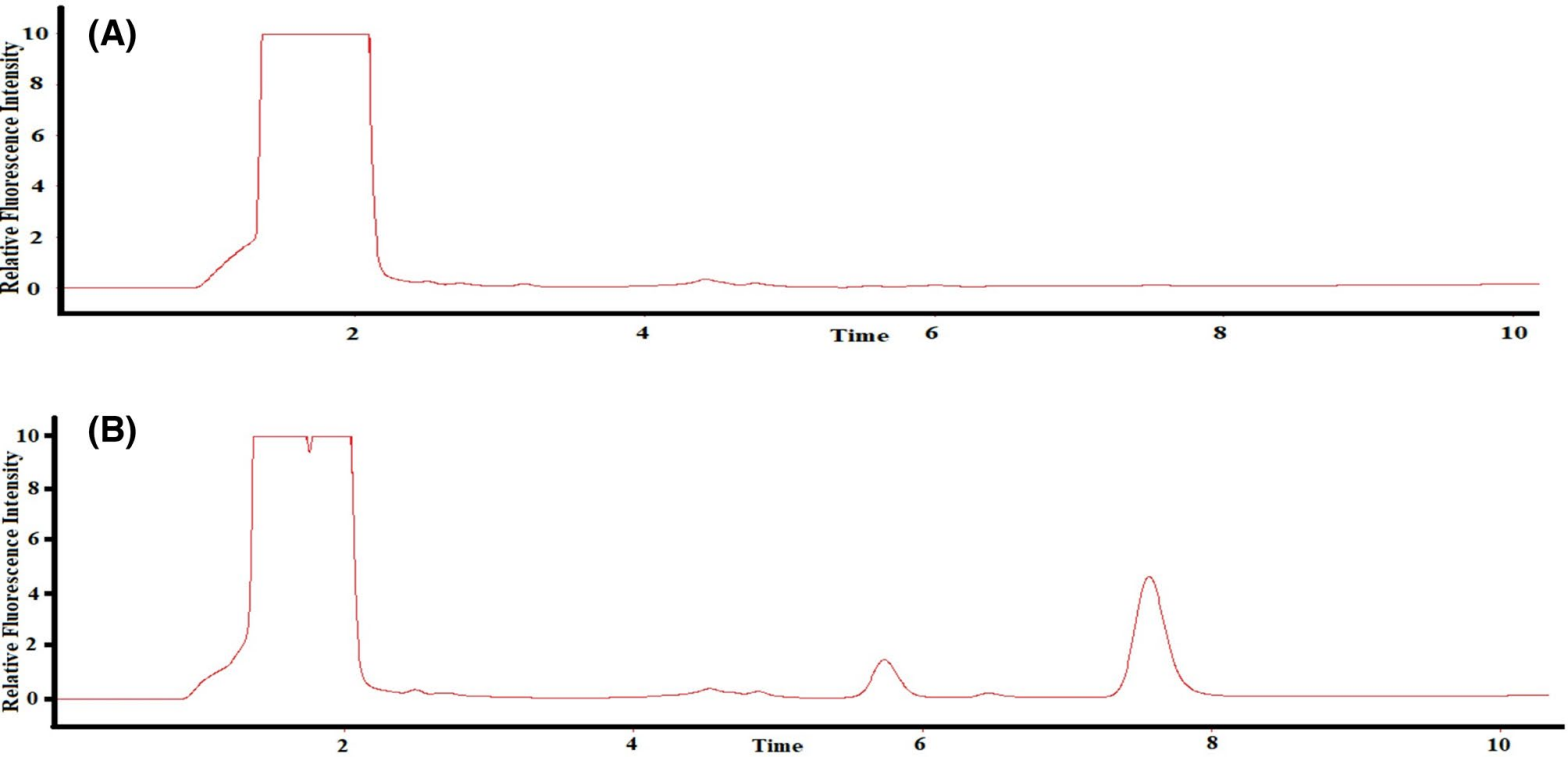
The chromatogram of (**A**) urine blank and (**B**) urine containing TAM (1.25 µg mL^−1^) and TTD (12.5 µg mL^−1^) using 280 nm and 350 nm as excitation and emission wavelengths, respectively.

A portion of 2.0 mL of urine was mixed with 1.0 mL of a standard solution containing TAM and TTD (concentration ranges: 1–15 µg mL^[-1^for TAM and 10–150 µg mL^[-1^ for TTD) and 6.0 mL of methanol. The mixture was vortex-mixed for 60 s and centrifuged at 4000 rpm for 10 min. The clear supernatant, which fell within the linear range of the proposed chromatographic method, was injected into the HPLC system. A blank sample was prepared similarly using drug-free urine, as shown in Fig. 5.

### Evaluation of system suitability

Six 20 µL injections of each standard solution mixture were made, and the resulting chromatograms were recorded. Key system suitability parameters, including column efficiency, plate number, retention time, and %RSD, were calculated. The results indicated high column efficiency and low standard deviations for the retention times of both TAM and TTD. The %RSD for the standard solution was found to be less than 2%. These findings are summarized in Table 2.

**Table 2:** Separation parameters for the proposed chromatographic method.

Parameter	Mixture 1	
	TAM	TTD
**Average retention time ± SD**	5.66 ± 0.02	7.26 ± 0.04
**Average N. of theoretical plates** ^**a**^	14,361	11,493
**Resolution** ^**b**^	6.95	
**RSD of peak area** ^**c**^	1.03	0.89

^a^ Theoretical plates < 2000 ^b^ Resolution < 1.5.

^c^ Number of determinations for the standard solution = 6.

## Results

### Method development

To achieve optimal separation of the drugs with short analysis time and symmetrical peak shapes, the composition of the mobile phase was carefully optimized. Various combinations of mobile phases (specifically the following ratios, 90:10, 80:20, 60:40, 50:50, 40:60, 20:80, and 10:90) were explored, including acetonitrile-water, methanol-water, acetonitrile-citrate buffer, methanol-citrate buffer, acetonitrile-phosphate buffer, and methanol-phosphate buffer in both isocratic and gradient modes. The influence of organic modifiers on separation efficiency was evaluated while keeping the buffer concentration and pH constant at 10 mM and 3.0, respectively. Other chromatographic parameters, such as column type (C_18_ and C_8_) and flow rate (1.0, 1.5, and 2.0 mL/min), were also investigated. It was found that gradient elution mode with the specific profile listed in Table 1 and consisted of three solvents: acetonitrile (A), water (B), and phosphate buffer (10 mM, pH 3.0) (C) gave the best separation parameters on C_18_ column with a flow rate of 1.0 mL/min. Fluorescence detection was used to measure RFI of the analytes, with excitation and emission wavelengths set at 280 nm and 350 nm, respectively.

### Method validation

ICH guidelines were followed to validate the suggested method^33^.

#### Linearity and range

To assess the linearity of the method, different concentration combinations, ranged from 0.1 to 1.5 and 1–15 for TAM and TTD, respectively, were injected in the gradient elution HPLC, with the specific gradient profile listed in Table 1. A linear relationship was observed between the area under the curve and the concentration of each drug. Linearity graphs for TAM and TTD were represented in Fig. 1S. The statistical data presented in Table 3, including low values for standard deviations of residuals, intercept, and slope, as well as high correlation coefficients, further confirms the linearity of the proposed method.

**Table 3:** Analytical parameters (*n* = 5) for the proposed chromatographic method.

Parameter	TAM	TTD
Linear range (µg mL ^−1^)	0.1– 1.5	1–15
Regression equation	Y = 14763.62X − 273.90	Y = 5249.30X − 1792.92
Slope	14763.62	5249.30
SD of slope (S_b_)	166.68	56.73
Intercept	−273.90	−1792.92
SD of intercept (S_a_)	141.097	480.19
Correlation coefficient	0.9996	0.9997
SD of residuals (S_y, x_)	220.01	748.74
LOD (µg mL^−1^)	0.03	0.30
LOQ (µg mL^−1^)	0.10	0.92

#### Limit of detection (LOD) and limit of quantification (LOQ)

LOD) and LOQ were calculated using the following equations: LOD = 3.3×Sa/ b ​and LOQ = 10×Sa/ b. where Sa is the standard deviation of the intercept and Sb is the slope of the calibration curve. The calculated LOD values for TAM and TTD were 0.03 and 0.30 µg mL^[-1^, respectively. The corresponding LOQ values were 0.10 and 0.92 µg mL^[-1^, respectively. These results are summarized in Table 3. These findings illustrate that the proposed method demonstrates heightened sensitivity in the analysis of TAM and TTD.

#### Precision and accuracy

To evaluate the precision of the method, the intra-day assay precision was assessed at three different concentration levels for both TAM and TTD. The concentrations tested were 0.15, 0.80, and 1.50 µg mL^[-1^for TAM and 1.50, 8.00, and 15.00 µg mL^[-1^ for TTD. The relative standard deviations (RSD) were calculated for each concentration level to quantify the precision of the method. These results are typically presented in Table 1S. To further evaluate the precision of the method, inter-day assay precision was evaluated at three different concentration levels for both TAM and TTD. The concentrations tested were 0.15, 0.80, and 1.50 µg mL^[-1^for TAM and 1.50, 8.00, and 15.00 µg mL^[-1^ for TTD. These analyses were conducted over three consecutive days to account for day-to-day variability. RSD was calculated for each concentration level and day to quantify the inter-day precision of the method. The results, typically presented in Table 1S, should indicate an RSD below 2%, signifying good inter-day precision.

To evaluate the accuracy of the method, a standard addition method was employed at three different concentration levels. Known amounts of TAM and TTD were added to samples, and the recovery of the added analytes was determined. The results, expressed as a percentage recovery, were documented in Table 2S. SD below 2% indicates that the method is accurate and reliable.

#### Robustness

To assess the robustness of the proposed method, small deliberate changes were made to the experimental parameters. Specifically, the flow rate of the mobile phase, excitation and emission wavelengths were slightly altered, and the impact on the method’s sensitivity was evaluated. The results of this robustness testing are presented in Table 3S.

## Discussions

TAM and TTD displayed indistinguishable fluorescence spectra, revealing emission peaks following excitation at 280 nm^15,21^. So, the fluorometric properties of both drugs were used to establish sensitive HPLC with fluorescence detection to separate and assay TAM and TTD. The chromatographic conditions were carefully optimized to achieve optimal separation of TAM and TTD, resulting in well-defined, symmetrical peaks with high theoretical plate numbers and resolution. The mobile phase composition, buffer type, and pH were adjusted to separate TAM and TTD within 5.66 and 7.26 min, respectively. This short analysis time makes the method suitable for routine analysis. The high accuracy and precision demonstrated by the method ensure reliable and reproducible results, making it suitable for quality control applications. Additionally, the robustness testing confirms the reliability of the method under slight variations in experimental conditions. Also, the selectivity of chromatographic methods enabled assaying of TAM and TTD in spiked human plasma and urine without any interference from plasma and urine components.

## Method application

### Pharmaceutical application

The developed method was successfully applied to analyze synthetic pharmaceutical formulations containing TAM and TTD. Statistical comparisons, including t-tests and F-tests, were conducted at a 95% confidence level to compare the results obtained from the proposed method with those of a previously reported method^21^. The calculated t-tests values were 0.80 and 0.42 for TAM and TTD, respectively. The calculated F-tests values were 1.67 and 2.77 for TAM and TTD, respectively. These results indicate that the proposed method is capable of accurately determining the drugs in pharmaceutical formulations, with no significant differences observed between the two methods. Table 4. summarizes the results of the statistical comparisons.

**Table 4.** Analysis of pharmaceutical formulation using the proposed chromatographic method.

Dosage form	Parameters	Reported method		Proposed method	
	Drug	TAM	TTD	TAM	TTD
TAM and TTD synthetic pharmaceutical formulation	% Recovery ^a^	99.30	100.96	99.73	100.76
	SD	0.96	0.93	0.74	0.56
	*t-*value^b^			0.80	0.42
	F-value ^b^			1.67	2.77

^a^ Mean of 5 determinations

^b^ Tabulated value at 95% confidence limit; t = 2.306 and F = 6.338.

### Spiked human plasma and urine application

Due to the high sensitivity and selectivity of the proposed chromatographic method, TAM and TTD levels in spiked human plasma and urine at various concentrations within the predetermined range could be assayed. The concentrations of TAM and TTD were determined using their respective regression equations: (RFI = 14763.62X − 273.90 for TAM and RFI = 5249.30X − 1792.92 for TTD). The observed mean recovery rates were (99.31–100.97%) ± (0.96–1.53) for TAM and (100.56–101.79%) ± (0.78–1.53) for TTD in human plasma, while in human urine, they were (98.71–101.55%) ± (0.58–1.44) for TAM and (99.53–101.84%) ± (0.34–1.31) for TTD (Table 5). These results demonstrate the method’s efficiency for quantifying TAM and TTD in spiked human plasma and urine samples.

**Table 5:** Determination of TAM and TTD in spiked human plasma and spiked human urine the proposed chromatographic method.

Conc. Level	% Recovery ± RSD ^a^			
µg mL^−1^	Spiked plasma		Spiked urine	
	Intra-day precision	Inter-day precision	Intra-day precision	Inter-day precision
**TAM**				
0.20	99.31 ± 0.96	99.46 ± 1.53	98.71 ± 1.03	99.38 ± 1.44
0.80	99.36 ± 1.14	100.64 ± 1.39	101.35 ± 0.92	101.55 ± 1.06
1.25	100.81 ± 1.20	100.97 ± 1.29	99.90 ± 0.58	99.59 ± 1.03
**TTD**				
2.00	101.41 ± 0.78	101.65 ± 1.20	99.93 ± 0.34	99.53 ± 1.31
8.00	101.29 ± 1.02	100.56 ± 1.27	101.08 ± 0.86	101.84 ± 1.04
12.50	101.39 ± 1.53	101.79 ± 1.41	100.44 ± 0.63	101.08 ± 0.89

^a^ Mean of three determinations.

### Evaluation of method of greenness

**Fig. 6:**
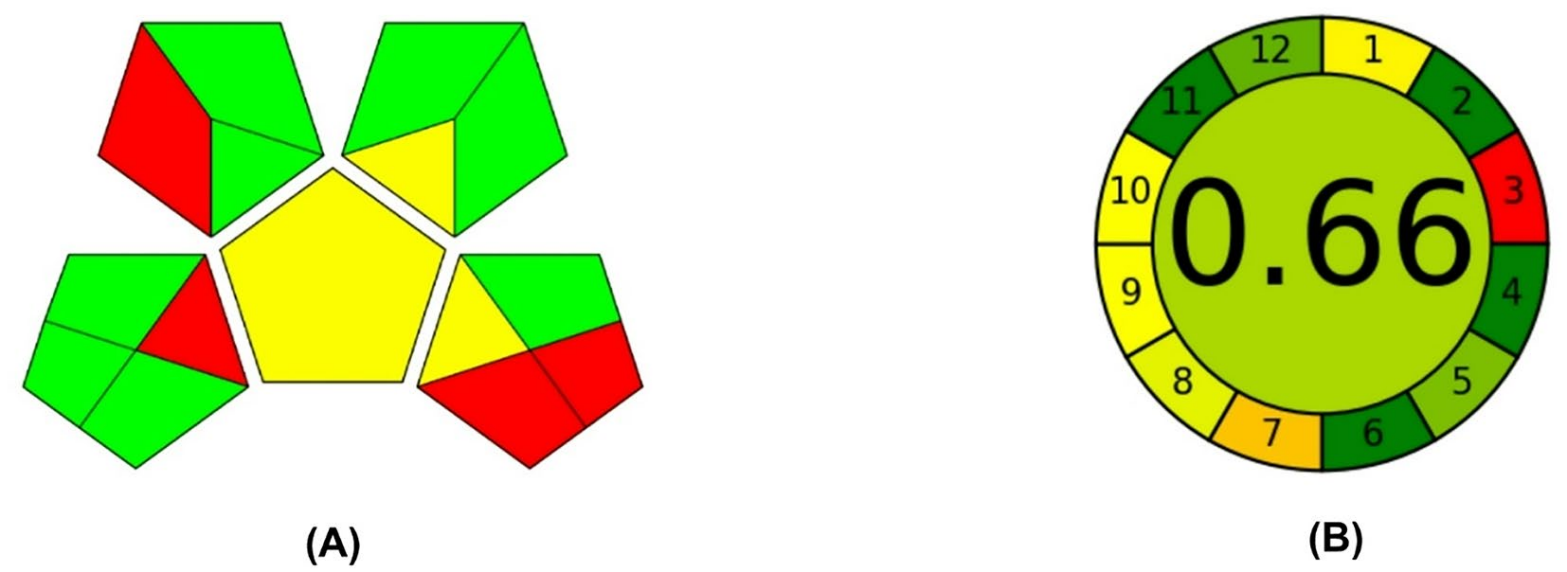
Greenness evaluation of the proposed chromatographic methods using (**A**) GAPI and (**B**) AGREE.

Analysts play a crucial role in protecting human health and the environment by ensuring the safe handling and disposal of harmful substances, particularly in industries like chemicals and pharmaceuticals^34^. Green analytical chemistry (GAC), which incorporates energy-efficient equipment and minimal waste generation, assists in lowering the quantities of hazardous chemicals and reagents. Current trends in the development of analytical methods include using fewer or non-toxic solvents, minimizing the use of devices for sample preparation, and using solventless extraction procedures^35,36^. To assess the environmental impact of analytical methods, tools like the Green Analytical Procedure Index (GAPI)^37^, and the Analytical Greenness Calculator (AGREE)^38^approaches are employed^39–41^. It is advised to compare analytical methodologies using a variety of evaluation tools in order to determine the ecological impact of each. GAPI provides a qualitative assessment of the environmental impact of each step of an analytical process, using a color-coded system (green, yellow, and red) to indicate low, medium, and high impact, respectively. The GAPI assessment of the proposed chromatographic method revealed a predominantly green profile, with 8 green, 3 yellow, and 4 red-shaded areas. This indicates a relatively low environmental impact (Fig. 6A).

AGREE is a quantitative tool that provides a numerical score and a visual representation of the method’s greenness. A higher score indicates a greener method. The AGREE assessment of the proposed method yielded a score close to 1 (0.66), indicating a high level of greenness. The pictogram also shows a predominantly green color, further emphasizing the method’s environmental friendliness (Fig. 6B). By employing both GAPI and AGREE, a comprehensive evaluation of the method’s greenness can be achieved. This information is valuable for selecting environmentally friendly analytical methods and minimizing the overall environmental impact of laboratory activities.

### Comparison with reported methods

The proposed chromatographic method was compared to several previously reported methods in terms of linear range, application, mobile phase, solvent, LOD, LOQ, and greenness evaluation. Table 6 provides a detailed comparison. It is evident that the proposed method, while having a relatively lower linear range compared to some other methods, is unique in its application to spiked human plasma and urine samples. Additionally, it is the only method that has been evaluated for its greenness, highlighting its environmental friendliness.

**Table 6:** A comparison between the proposed and the reported methods.

Method	Linear range (µg mL^−1^)		Mobile phase/ Solvent	LOD (µg mL^−1^)		LOQ (µg mL^−1^)		Application	Greenness evaluation
	TAM	TTD		TAM	TTD	TAM	TTD		
**Spectrophotometry** ^20^	5.0–25.0	10.0–50.0	methanol	N.A.	N.A.	N.A.	N.A.	dosage form	N.A.
**Spectrofluorimetry** ^21^	0.75–3.50	2.5–20.0	double distilled water	0.17	0.68	0.52	2.04	dosage form	N.A.
**HPLC-UV** ^22^	0.2–0.6	2.0–6.0	Acetonitrile: 20mM ammonium acetate buffer (pH 7.8) (60: 40, v/v)	0.004	0.004	0.013	0.011	dosage form	N.A.
**HPLC-UV** ^23^	2.0–6.0	20.0–60.0	phosphate buffer (pH 4.0): Methanol (60:40, v/v)	0.89	2.92	2.70	8.84	dosage form/ Stability indicating assay	N.A.
**HPTLC** ^24^	16.0–64.0	160.0–640.0	methanol: ethyl acetate: triethylamine in the ratio (5:5:0.3, v/v/v)	4.42	7.48	14.78	24.95	dosage form	N.A.
**HPTLC** ^25^	10.0–70.0	40.0–400.0	ethyl acetate: methanol: ammonia (6:4:0.05, v/v/v)	3.0	12.0	10.0	40.0	dosage form	N.A.
**HPTLC** ^26^	2.0–40.0	20.0–180.0	ethyl acetate: n-hexane: diethylamine (9:3:1, v/v/v)	0.52	4.38	1.6	13.3	dosage form/ Stability indicating assay	N.A.
**Proposed method**	0.1–1.5	1.0–15.0	acetonitrile, water, and phosphate buffer (10 mM, pH 3.0) in a gradient elution	0.03	0.30	0.10	0.92	dosage form/ spiked plasma and urine	Applied to GAPI and AGREE

* N.A (not applicable).

## Conclusion

A novel, green, sensitive, and accurate reversed-phase chromatographic method with fluorescence detection has been developed for the simultaneous determination of TAM and TTD. The method was validated according to ICH. Linear calibration curves were attained over the concentration ranges of 0.1–1.5 µg mL^[-1^for TAM and 1–15 µg mL^[-1^for TTD. The calculated LOD values for TAM and TTD were 0.03 and 0.30 µg mL^[-1^, respectively. The corresponding LOQ values were 0.10 and 0.92 µg mL^[-1^, respectively. The method was successfully applied to analyze TAM and TTD in pharmaceutical formulations and spiked human plasma and urine samples. The observed mean recovery percent were 99.31–100.97% for TAM and 100.56–101.79% for TTD in human plasma, while in human urine, they were 98.71–101.55% for TAM and 99.53–101.84% for TTD. The high accuracy and precision demonstrated by the method make it suitable for quality control applications. By incorporating green analytical chemistry principles, the method was assessed using the GAPI and AGREE tools, confirming its environmental friendliness. The main limitation of the proposed HPLC method is the need for proficient personnel.

## Electronic supplementary material

Below is the link to the electronic supplementary material.

Supplementary Material 1

## Data Availability

All data generated or analyzed during this study are included in this published article.
