# Supplementary material for: Green HPLC-Fluorescence detection method for concurrent analysis of Tamsulosin hydrochloride and Tolterodine tartrate in dosage forms and biological fluids
Source: Sci Rep. 2025 Mar 27;15:10615. doi: 10.1038/s41598-025-92183-6 (PMC11950346; doi:10.1038/s41598-025-92183-6)
Supplement: Supplementary file 1 — Supplementary Material 1 [file 41598_2025_92183_MOESM1_ESM.docx]

**Green HPLC-Fluorescence Detection Method for Concurrent Analysis of Tamsulosin Hydrochloride and Tolterodine Tartrate in Dosage Forms and Biological Fluids**

Sayed M. Derayea ^a^, Khalid M. Badr El-Din ^a^, Ahmed S. Ahmed ^b*^, Mohamed Oraby ^b, c^, Mohamed A. Abdelshakour ^b^.

*^a^ Department of Pharmaceutical Analytical Chemistry, Faculty of Pharmacy, Minia University, Minia 61519, Egypt.*

*^b^ Department of Pharmaceutical Analytical Chemistry, Faculty of Pharmacy, Sohag University, Sohag 82524, Egypt.*

*^c^ Pharmaceutical Chemistry Department, College of Pharmacy, Al-Esraa University, Baghdad 10069, Iraq.*

**Corresponding authors: Ahmed S. Ahmed,* **ahmed.saad@pharm.sohag.edu.eg**

**Table 1 S**: Evaluation of the intra-day and inter-day precision of the proposed chromatographic method.

| **Method** | **Conc. level** | **% Recovery ± RSD** ^a^ | |
| --- | --- | --- | --- |
|  | **μg mL^-1^** | **Intra-day precision** | **Inter-day precision** |
| **TAM** | 0.15 | 101.25 ± 0.63 | 100.79 ± 1.39 |
|  | 0.80 | 99.82 ± 0.94 | 99.72 ± 1.05 |
|  | 1.50 | 99.70 ± 1.09 | 99.83 ± 1.37 |
| **TTD** | 1.50 | 100.61 ± 0.25 | 101.22 ± 0.78 |
|  | 8.00 | 99.96 ± 0.82 | 99.92 ± 1.26 |
|  | 15.00 | 99.46 ± 0.72 | 99.88 ± 1.00 |
| ^a^ Mean of three determinations. | | | |

**Table 2 S**: Accuracy of the proposed chromatographic method using the standard addition method.

|  | **Amount taken**  **μg mL^-1^** | **Amount added**  **μg mL^-1^** | **Amount found**  **μg mL^-1^** | **% Recovery ± SD**^a^ |
| --- | --- | --- | --- | --- |
|  | **(TAM)** |  |  |  |
|  | 0.15 | 0 | 0.15 | 100.55 ± 0.55 |
| **TAM and TTD** **synthetic pharmaceutical formulation** | 0.15 | 0.15 | 0.30 | 99.22 ± 1.11 |
|  | 0.15 | 0.85 | 1.01 | 100.68 ± 1.09 |
|  | 0.15 | 1.35 | 1.49 | 99.54 ± 0.55 |
|  | **(TTD)** |  |  |  |
|  | 1.50 | 0 | 1.53 | 101.64 ± 0.53 |
|  | 1.50 | 1.50 | 3.03 | 100.91 ± 0.97 |
|  | 1.50 | 8.50 | 10.11 | 101.07 ± 0.80 |
|  | 1.50 | 13.50 | 14.99 | 99.90 ± 0.19 |
| ^a^ Mean of three determinations. | | | | |

**Table 3 S:** Robustness of the proposed chromatographic method.

| **Parameter** | | **% Recovery ± SD ^a^** | |
| --- | --- | --- | --- |
|  |  | **TAM ^b^** | **TTD ^b^** |
| Flow rate (mL) | 0.98 | 99.13 ± 1.26 | 99.77 ± 0.45 |
|  | 1.02 | 99.43 ± 0.79 | 99.95 ± 1.10 |
| Excitation wavelength (nm) | 278 | 98.95 ± 0.73 | 99.10 ± 0.30 |
|  | 282 | 100.26 ± 0.89 | 99.86 ± 0.25 |
| Emission wavelength (nm) | 348 | 98.65 ± 1.51 | 101.36 ± 0.48 |
|  | 352 | 99.83 ± 1.08 | 99.24 ± 0.35 |
| ^a^ Mean of three determinations.  ^b^ Drug concentrations are 0.8 μg mL^-1^ for TAM and 8.0 μg mL^-1^ for TTD. | | | |

**Fig. 1 S:** Linearity graphs for (A) TAM and (B) TTD
